# (4-Oxido-2-oxo-1,2-dihydro­pyrimidine-5-carboxyl­ato-κ^2^ *O* ^4^,*O* ^5^)(4-oxido-2-oxo-1,2-dihydro­pyrimidin-3-ium-5-carboxyl­ato-κ^2^ *O* ^4^,*O* ^5^)bis­(1,10-phenanthroline-κ^2^ *N*,*N*′)gadolinium(III) dihydrate

**DOI:** 10.1107/S1600536808025713

**Published:** 2008-08-16

**Authors:** Wei Xiong, Huihui Xing, Yan Su, Zilu Chen

**Affiliations:** a College of Chemistry and Chemical Engineering, Guangxi Normal University, Yucai Road 15, Guilin 541004, People’s Republic of China

## Abstract

The title compound, [Gd(C_5_H_2_N_2_O_4_)(C_5_H_3_N_2_O_4_)(C_12_H_8_N_2_)_2_]·2H_2_O, was obtained from a solvothermal reaction of 2,4-dihydroxy­pyrimidine-5-carboxylic acid (H_3_iso), GdCl_3_·6H_2_O and 1,10-phenanthroline (phen). The Gd^III^ ion is located on a twofold rotation axis and is coordinated by four N atoms from two chelating phen ligands and four O atoms (5-carboxyl­ate and 4-oxido O atoms) from H_2_iso^−^ and Hiso^2−^ ligands. The mol­ecules are linked into a three-dimensional network by N—H⋯O, N—H⋯N and O—H⋯O hydrogen bonds. The H atom involved in an N—H⋯N hydrogen bond is disordered around a twofold rotation axis with half occupancy.

## Related literature

For isostructural lanthanide complexes with 2,4-dioxo-1,2,3,4-tetra­hydro­pyrimidine-5-carboxylic acid, see: Sun & Jin (2004*a* ▶,*b* ▶); Xing *et al.* (2008*a* ▶). For related literature, see: Hueso-Ureña *et al.* (1993 ▶, 1996 ▶); Baran *et al.* (1996 ▶); Luo *et al.* (2002 ▶); Maistralis *et al.* (1991 ▶, 1992 ▶); Xing *et al.* (2008*b* ▶).
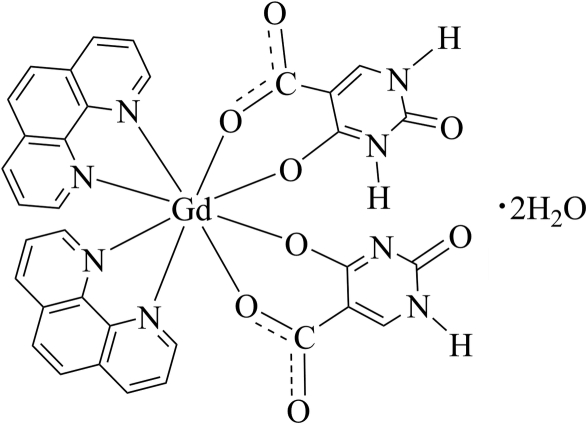


## Experimental

### 

#### Crystal data

[Gd(C_5_H_2_N_2_O_4_)(C_5_H_3_N_2_O_4_)(C_12_H_8_N_2_)_2_]·2H_2_O
*M* *_r_* = 862.87
Monoclinic, 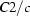

*a* = 17.158 (8) Å
*b* = 14.504 (7) Å
*c* = 13.197 (7) Å
β = 99.955 (5)°
*V* = 3235 (3) Å^3^
*Z* = 4
Mo *K*α radiation
μ = 2.13 mm^−1^
*T* = 273 (2) K
0.20 × 0.10 × 0.10 mm

#### Data collection

Bruker APEXII CCD area-detector diffractometer
Absorption correction: multi-scan (*SADABS*; Bruker, 1998 ▶) *T*_min_ = 0.676, *T*_max_ = 0.816
9884 measured reflections
3686 independent reflections
3212 reflections with *I* > 2σ(*I*)
*R*_int_ = 0.039

#### Refinement

*R*[*F* ^2^ > 2σ(*F* ^2^)] = 0.031
*wR*(*F* ^2^) = 0.064
*S* = 1.05
3686 reflections
240 parameters
H-atom parameters constrained
Δρ_max_ = 0.74 e Å^−3^
Δρ_min_ = −0.65 e Å^−3^

### 

Data collection: *APEX2* (Bruker, 2004 ▶); cell refinement: *APEX2*; data reduction: *SAINT* (Bruker, 2004 ▶); program(s) used to solve structure: *SHELXS97* (Sheldrick, 2008 ▶); program(s) used to refine structure: *SHELXL97* (Sheldrick, 2008 ▶); molecular graphics: *SHELXTL* (Sheldrick, 2008 ▶); software used to prepare material for publication: *SHELXTL*.

## Supplementary Material

Crystal structure: contains datablocks global, I. DOI: 10.1107/S1600536808025713/ci2644sup1.cif

Structure factors: contains datablocks I. DOI: 10.1107/S1600536808025713/ci2644Isup2.hkl

Additional supplementary materials:  crystallographic information; 3D view; checkCIF report

## Figures and Tables

**Table:** 

Gd1—O1	2.288 (2)
Gd1—O3	2.344 (2)
Gd1—N3	2.586 (3)
Gd1—N4	2.603 (3)

**Table:** 

O1—Gd1—O1^i^	88.22 (11)
O1—Gd1—O3^i^	82.54 (8)
O1—Gd1—O3	73.65 (8)
O3^i^—Gd1—O3	146.71 (11)
O1—Gd1—N3^i^	148.84 (8)
O3—Gd1—N3^i^	135.03 (8)
O1—Gd1—N3	105.26 (9)
O3—Gd1—N3	74.86 (8)
O1—Gd1—N4	80.80 (8)
O3—Gd1—N4	122.38 (8)
N3—Gd1—N4	63.39 (8)
O1—Gd1—N4^i^	147.64 (8)
O3—Gd1—N4^i^	74.79 (8)
N3—Gd1—N4^i^	72.83 (8)
N4—Gd1—N4^i^	123.04 (11)

Symmetry code: (i) 
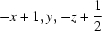
.

**Table 2:** Hydrogen-bond geometry (Å, °)

*D*—H⋯*A*	*D*—H	H⋯*A*	*D*⋯*A*	*D*—H⋯*A*
N1—H1⋯O2^ii^	0.86	2.04	2.898 (3)	178
N1—H1⋯O1^ii^	0.86	2.60	3.160 (4)	124
N2—H2⋯N2^iii^	0.86	1.81	2.669 (5)	174
O5—H5*A*⋯O4^iv^	0.85	2.14	2.970 (4)	164
O5—H5*B*⋯O2^v^	0.85	2.14	2.985 (4)	173

Symmetry codes: (ii) 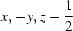; (iii) 
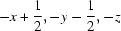
; (iv) 
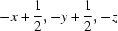
; (v) 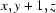.

**Fig. 1.**
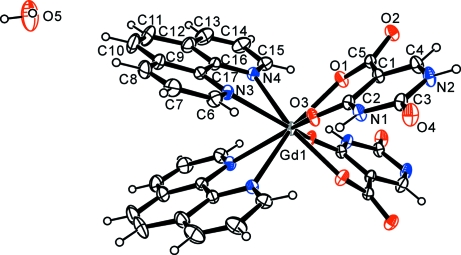
A view of the molecular structure of (I) with the atom-numbering scheme. Displacement ellipsoids are drawn at the 30% probability level. Unlabelled atoms are related to labelled atoms by the symmetry operation (1-x, y, 1/2-z).

## supplementary crystallographic information

### Comment

2,4-Dihydroxypyrimidine-5-carboxylic acid has been extensively used in the preparation of metal complexes because of its versatile coordination modes. It can connect metal ions to form robust networks or some porous coordination polymers. Though various transition metal complexes with 2,4-dihydroxypyrimidine-5-carboxylate have been reported (Maistralis *et al.*, 1991, 1992; Hueso-Ureña *et al.*, 1993, 1996; Baran *et al.*, 1996; Luo *et al.*, 2002; Sun & Jin 2004*a*), lanthanide complexes are very limited. Only lanthanide complexes of Yb^III^, Tb^III^, Pr^III^, Eu^III^, Nd^III^ and Er^III^ have been reported (Sun & Jin 2004*b*; Xing *et al.*,2008*a*,*b*). In this paper, we report a new Gd^III^ complex, Gd(Hiso)(H_2_iso)(phen)_2_.2H_2_O, (I).

In compound (I), the Gd^III^ atom is located on a twofold rotation axis and coordinated in a square antiprismatic geometry by four N atoms belonging to two chelating phen ligands and four O atoms from one monovalent and one divalent 2,4-dihydroxypyrimidine-5-carboxylate anions. The Gd—O bond lengths [2.288 (2) and 2.334 (2) Å] are shorter than the Gd—N bond lengths [2.586 (3) and 2.603 (3) Å].

The molecules are linked into a three-dimensional network by N—H···O, N—H···N and O—H···O hydrogen bonds (Table 2).

### Experimental

A mixture of 2,4-dihydroxypyrimidine-5-carboxylic acid (0.0312 g, 0.2 mmol), GdCl_3_.6H_2_O (0.0743 g, 0.2 mmol), phen.H_2_O (0.0396 g, 0.2 mmol), NaOH (0.008 g, 0.2 mmol) and water (15 ml) was sealed in a 25 ml Teflon-lined stainless-steel reactor and heated at 383 K for 120 h. It was then cooled over a period of 48 h, light yellow crystals were isolated in 80% yield. Elemental analysis for C_34_H_25_GdN_8_O_10_, calculated: C 47.32, H 2.92, N 12.98%; found: C 47.59,H 2.96, N 13.24%.

### Refinement

H atoms of the water molecule were located in a difference Fourier map and allowed to ride on the O atom with *U*_iso_(H) = 1.5*U*_eq_(O). The remaining H atoms were placed at calculated positions (C—H = 0.93 Å and N—H = 0.86 Å) and were included in the refinement in the riding model approximation, with *U*_iso_(H) = 1.2*U*_eq_(C, N).

### Crystal data

**Table:**

[Gd(C_5_H_2_N_2_O_4_)(C_5_H_3_N_2_O_4_)(C_12_H_8_N_2_)_2_]·2H_2_O	*F*_000_ = 1716
*M**_r_* = 862.87	*D*_x_ = 1.772 Mg m^−^^3^
Monoclinic, *C*2/*c*	Mo *K*α radiation λ = 0.71073 Å
Hall symbol: -C 2yc	Cell parameters from 3284 reflections
*a* = 17.158 (8) Å	θ = 2.3–25.5º
*b* = 14.504 (7) Å	µ = 2.13 mm^−^^1^
*c* = 13.197 (7) Å	*T* = 273 (2) K
β = 99.955 (5)º	Block, colourless
*V* = 3235 (3) Å^3^	0.20 × 0.10 × 0.10 mm
*Z* = 4	

### Data collection

**Table:**

Bruker APEXII CCD area-detector diffractometer	3686 independent reflections
Radiation source: fine-focus sealed tube	3212 reflections with *I* > 2σ(*I*)
Monochromator: graphite	*R*_int_ = 0.039
*T* = 273(2) K	θ_max_ = 27.5º
φ and ω scans	θ_min_ = 2.3º
Absorption correction: multi-scan(SADABS; Bruker, 1998)	*h* = −21→22
*T*_min_ = 0.676, *T*_max_ = 0.816	*k* = −18→13
9884 measured reflections	*l* = −17→16

### Refinement

**Table:**

Refinement on *F*^2^	Secondary atom site location: difference Fourier map
Least-squares matrix: full	Hydrogen site location: inferred from neighbouring sites
*R*[*F*^2^ > 2σ(*F*^2^)] = 0.031	H-atom parameters constrained
*wR*(*F*^2^) = 0.064	*w* = 1/[σ^2^(*F*_o_^2^) + (0.0258*P*)^2^] where *P* = (*F*_o_^2^ + 2*F*_c_^2^)/3
*S* = 1.05	(Δ/σ)_max_ = 0.001
3686 reflections	Δρ_max_ = 0.74 e Å^−^^3^
240 parameters	Δρ_min_ = −0.65 e Å^−^^3^
Primary atom site location: structure-invariant direct methods	Extinction correction: none

### Special details

**Table:**

Geometry. All e.s.d.'s (except the e.s.d. in the dihedral angle between two l.s. planes) are estimated using the full covariance matrix. The cell e.s.d.'s are taken into account individually in the estimation of e.s.d.'s in distances, angles and torsion angles; correlations between e.s.d.'s in cell parameters are only used when they are defined by crystal symmetry. An approximate (isotropic) treatment of cell e.s.d.'s is used for estimating e.s.d.'s involving l.s. planes.
Refinement. Refinement of *F*^2^ against ALL reflections. The weighted *R*-factor *wR* and goodness of fit *S* are based on *F*^2^, conventional *R*-factors *R* are based on *F*, with *F* set to zero for negative *F*^2^. The threshold expression of *F*^2^ > σ(*F*^2^) is used only for calculating *R*-factors(gt) *etc*. and is not relevant to the choice of reflections for refinement. *R*-factors based on *F*^2^ are statistically about twice as large as those based on *F*, and *R*- factors based on ALL data will be even larger.

### Fractional atomic coordinates and isotropic or equivalent isotropic displacement parameters (Å^2^)

**Table:**

	*x*	*y*	*z*	*U*_iso_*/*U*_eq_	Occ. (<1)
Gd1	0.5000	0.111515 (14)	0.2500	0.02254 (7)	
O1	0.42801 (12)	−0.00176 (14)	0.31169 (16)	0.0329 (5)	
O2	0.34603 (14)	−0.11272 (15)	0.33997 (16)	0.0352 (5)	
O3	0.40327 (12)	0.06523 (14)	0.11160 (16)	0.0320 (5)	
O4	0.26936 (15)	−0.11830 (15)	−0.13464 (17)	0.0424 (6)	
N1	0.33231 (15)	−0.02639 (17)	−0.00642 (19)	0.0296 (6)	
H1	0.3371	0.0157	−0.0508	0.035*	
N2	0.28238 (16)	−0.16877 (18)	0.0304 (2)	0.0361 (7)	
H2	0.2589	−0.2201	0.0125	0.043*	0.50
N3	0.40482 (14)	0.25042 (17)	0.2136 (2)	0.0300 (6)	
N4	0.46494 (14)	0.19709 (17)	0.40957 (19)	0.0282 (6)	
C1	0.34878 (17)	−0.0740 (2)	0.1674 (2)	0.0248 (7)	
C2	0.36429 (17)	−0.0080 (2)	0.0939 (2)	0.0251 (7)	
C3	0.29303 (19)	−0.1061 (2)	−0.0434 (2)	0.0304 (7)	
C4	0.3080 (2)	−0.1512 (2)	0.1292 (2)	0.0348 (8)	
H4	0.2973	−0.1949	0.1765	0.042*	
C5	0.37526 (17)	−0.0627 (2)	0.2800 (2)	0.0248 (6)	
C6	0.37014 (18)	0.2746 (2)	0.1201 (3)	0.0372 (8)	
H6	0.3689	0.2319	0.0672	0.045*	
C7	0.3352 (2)	0.3608 (3)	0.0960 (3)	0.0475 (10)	
H7	0.3113	0.3747	0.0290	0.057*	
C8	0.3372 (2)	0.4239 (3)	0.1725 (3)	0.0499 (10)	
H8	0.3157	0.4822	0.1577	0.060*	
C9	0.3710 (2)	0.4014 (2)	0.2728 (3)	0.0428 (9)	
C10	0.3742 (2)	0.4633 (3)	0.3580 (4)	0.0592 (12)	
H10	0.3547	0.5229	0.3461	0.071*	
C11	0.4042 (3)	0.4380 (3)	0.4535 (4)	0.0618 (12)	
H11	0.4056	0.4803	0.5068	0.074*	
C12	0.4346 (2)	0.3467 (3)	0.4763 (3)	0.0442 (9)	
C13	0.4638 (2)	0.3150 (3)	0.5755 (3)	0.0577 (11)	
H13	0.4648	0.3543	0.6314	0.069*	
C14	0.4906 (2)	0.2274 (3)	0.5910 (3)	0.0510 (10)	
H14	0.5088	0.2056	0.6571	0.061*	
C15	0.49028 (19)	0.1703 (3)	0.5054 (2)	0.0359 (8)	
H15	0.5089	0.1103	0.5166	0.043*	
C16	0.43530 (18)	0.2836 (2)	0.3944 (2)	0.0298 (7)	
C17	0.40366 (18)	0.3123 (2)	0.2911 (3)	0.0317 (7)	
O5	0.3084 (2)	0.6863 (2)	0.3396 (2)	0.0901 (11)	
H5A	0.2777	0.6708	0.2847	0.135*	
H5B	0.3166	0.7439	0.3441	0.135*	

### Atomic displacement parameters (Å^2^)

**Table:**

	*U*^11^	*U*^22^	*U*^33^	*U*^12^	*U*^13^	*U*^23^
Gd1	0.02861 (12)	0.01718 (12)	0.01966 (12)	0.000	−0.00192 (8)	0.000
O1	0.0440 (13)	0.0318 (13)	0.0208 (11)	−0.0131 (11)	−0.0007 (10)	0.0008 (9)
O2	0.0496 (14)	0.0348 (13)	0.0209 (11)	−0.0118 (11)	0.0055 (10)	0.0019 (10)
O3	0.0427 (13)	0.0250 (12)	0.0243 (12)	−0.0113 (10)	−0.0055 (10)	0.0032 (9)
O4	0.0627 (16)	0.0363 (14)	0.0217 (12)	−0.0031 (12)	−0.0108 (11)	−0.0055 (10)
N1	0.0409 (15)	0.0272 (15)	0.0179 (13)	−0.0076 (12)	−0.0023 (11)	0.0023 (11)
N2	0.0533 (18)	0.0244 (15)	0.0264 (15)	−0.0114 (13)	−0.0043 (13)	−0.0024 (12)
N3	0.0299 (14)	0.0263 (15)	0.0319 (15)	0.0024 (11)	0.0000 (12)	0.0012 (11)
N4	0.0324 (14)	0.0271 (14)	0.0244 (14)	0.0016 (11)	0.0028 (11)	0.0010 (11)
C1	0.0306 (16)	0.0229 (16)	0.0197 (15)	−0.0035 (13)	0.0005 (13)	0.0026 (12)
C2	0.0286 (16)	0.0229 (16)	0.0215 (16)	0.0005 (13)	−0.0022 (13)	−0.0024 (12)
C3	0.0350 (17)	0.0259 (17)	0.0268 (17)	0.0014 (14)	−0.0045 (14)	−0.0039 (14)
C4	0.049 (2)	0.0291 (18)	0.0247 (17)	−0.0116 (16)	0.0025 (15)	0.0013 (14)
C5	0.0331 (17)	0.0219 (16)	0.0191 (15)	−0.0014 (13)	0.0031 (13)	−0.0010 (12)
C6	0.0365 (18)	0.039 (2)	0.0339 (19)	0.0071 (16)	−0.0002 (15)	0.0067 (15)
C7	0.043 (2)	0.051 (2)	0.047 (2)	0.0128 (18)	0.0026 (18)	0.0207 (19)
C8	0.049 (2)	0.033 (2)	0.068 (3)	0.0107 (18)	0.010 (2)	0.019 (2)
C9	0.040 (2)	0.028 (2)	0.062 (3)	0.0083 (15)	0.0133 (18)	0.0040 (17)
C10	0.069 (3)	0.030 (2)	0.079 (3)	0.016 (2)	0.015 (2)	−0.006 (2)
C11	0.077 (3)	0.037 (2)	0.071 (3)	0.014 (2)	0.013 (3)	−0.025 (2)
C12	0.050 (2)	0.039 (2)	0.045 (2)	0.0016 (18)	0.0112 (18)	−0.0138 (18)
C13	0.063 (3)	0.068 (3)	0.043 (2)	0.007 (2)	0.010 (2)	−0.023 (2)
C14	0.054 (2)	0.069 (3)	0.029 (2)	0.012 (2)	0.0063 (18)	−0.0081 (19)
C15	0.0372 (18)	0.043 (2)	0.0270 (18)	0.0053 (16)	0.0051 (15)	0.0013 (15)
C16	0.0294 (17)	0.0257 (17)	0.0348 (19)	0.0005 (13)	0.0067 (14)	−0.0027 (14)
C17	0.0271 (16)	0.0278 (18)	0.041 (2)	0.0021 (13)	0.0089 (15)	0.0027 (15)
O5	0.133 (3)	0.051 (2)	0.070 (2)	0.000 (2)	−0.027 (2)	0.0100 (16)

### Geometric parameters (Å, °)

**Table:**

Gd1—O1	2.288 (2)	C1—C5	1.485 (4)
Gd1—O1^i^	2.288 (2)	C4—H4	0.93
Gd1—O3^i^	2.344 (2)	C6—C7	1.399 (5)
Gd1—O3	2.344 (2)	C6—H6	0.93
Gd1—N3^i^	2.586 (3)	C7—C8	1.358 (6)
Gd1—N3	2.586 (3)	C7—H7	0.93
Gd1—N4	2.603 (3)	C8—C9	1.389 (5)
Gd1—N4^i^	2.603 (3)	C8—H8	0.93
O1—C5	1.282 (3)	C9—C17	1.414 (4)
O2—C5	1.242 (3)	C9—C10	1.432 (6)
O3—C2	1.256 (3)	C10—C11	1.329 (6)
O4—C3	1.216 (4)	C10—H10	0.93
N1—C2	1.369 (4)	C11—C12	1.435 (6)
N1—C3	1.383 (4)	C11—H11	0.93
N1—H1	0.86	C12—C13	1.396 (5)
N2—C4	1.327 (4)	C12—C16	1.417 (5)
N2—C3	1.367 (4)	C13—C14	1.355 (5)
N2—H2	0.86	C13—H13	0.93
N3—C6	1.322 (4)	C14—C15	1.399 (5)
N3—C17	1.364 (4)	C14—H14	0.93
N4—C15	1.323 (4)	C15—H15	0.93
N4—C16	1.356 (4)	C16—C17	1.439 (4)
C1—C4	1.369 (4)	O5—H5A	0.85
C1—C2	1.421 (4)	O5—H5B	0.85
			
O1—Gd1—O1^i^	88.22 (11)	N1—C2—C1	116.0 (3)
O1—Gd1—O3^i^	82.54 (8)	O4—C3—N2	123.0 (3)
O1^i^—Gd1—O3^i^	73.65 (8)	O4—C3—N1	122.0 (3)
O1—Gd1—O3	73.65 (8)	N2—C3—N1	114.9 (3)
O1^i^—Gd1—O3	82.54 (8)	N2—C4—C1	125.5 (3)
O3^i^—Gd1—O3	146.71 (11)	N2—C4—H4	117.3
O1—Gd1—N3^i^	148.84 (8)	C1—C4—H4	117.3
O1^i^—Gd1—N3^i^	105.26 (9)	O2—C5—O1	122.3 (3)
O3^i^—Gd1—N3^i^	74.86 (8)	O2—C5—C1	119.0 (3)
O3—Gd1—N3^i^	135.03 (8)	O1—C5—C1	118.6 (3)
O1—Gd1—N3	105.26 (9)	N3—C6—C7	123.7 (3)
O1^i^—Gd1—N3	148.84 (8)	N3—C6—H6	118.1
O3^i^—Gd1—N3	135.03 (8)	C7—C6—H6	118.1
O3—Gd1—N3	74.86 (8)	C8—C7—C6	118.6 (4)
N3^i^—Gd1—N3	77.63 (12)	C8—C7—H7	120.7
O1—Gd1—N4	80.80 (8)	C6—C7—H7	120.7
O1^i^—Gd1—N4	147.64 (8)	C7—C8—C9	120.2 (4)
O3^i^—Gd1—N4	74.79 (8)	C7—C8—H8	119.9
O3—Gd1—N4	122.38 (8)	C9—C8—H8	119.9
N3^i^—Gd1—N4	72.83 (8)	C8—C9—C17	117.7 (4)
N3—Gd1—N4	63.39 (8)	C8—C9—C10	123.7 (4)
O1—Gd1—N4^i^	147.64 (8)	C17—C9—C10	118.6 (4)
O1^i^—Gd1—N4^i^	80.80 (8)	C11—C10—C9	121.9 (4)
O3^i^—Gd1—N4^i^	122.38 (8)	C11—C10—H10	119.1
O3—Gd1—N4^i^	74.79 (8)	C9—C10—H10	119.1
N3^i^—Gd1—N4^i^	63.39 (8)	C10—C11—C12	121.4 (4)
N3—Gd1—N4^i^	72.83 (8)	C10—C11—H11	119.3
N4—Gd1—N4^i^	123.04 (11)	C12—C11—H11	119.3
C5—O1—Gd1	140.60 (19)	C13—C12—C16	116.9 (3)
C2—O3—Gd1	132.05 (19)	C13—C12—C11	124.0 (4)
C2—N1—C3	126.4 (3)	C16—C12—C11	119.1 (4)
C2—N1—H1	116.8	C14—C13—C12	120.5 (4)
C3—N1—H1	116.8	C14—C13—H13	119.7
C4—N2—C3	120.6 (3)	C12—C13—H13	119.7
C4—N2—H2	119.7	C13—C14—C15	118.7 (4)
C3—N2—H2	119.7	C13—C14—H14	120.7
C6—N3—C17	117.6 (3)	C15—C14—H14	120.7
C6—N3—Gd1	123.3 (2)	N4—C15—C14	123.6 (3)
C17—N3—Gd1	117.6 (2)	N4—C15—H15	118.2
C15—N4—C16	117.7 (3)	C14—C15—H15	118.2
C15—N4—Gd1	123.4 (2)	N4—C16—C12	122.5 (3)
C16—N4—Gd1	117.1 (2)	N4—C16—C17	118.5 (3)
C4—C1—C2	116.3 (3)	C12—C16—C17	119.0 (3)
C4—C1—C5	120.5 (3)	N3—C17—C9	122.0 (3)
C2—C1—C5	123.2 (3)	N3—C17—C16	118.0 (3)
O3—C2—N1	117.1 (3)	C9—C17—C16	120.0 (3)
O3—C2—C1	126.9 (3)	H5A—O5—H5B	113.1
			
O1^i^—Gd1—O1—C5	−72.3 (3)	C4—N2—C3—O4	−178.6 (3)
O3^i^—Gd1—O1—C5	−146.0 (3)	C4—N2—C3—N1	0.8 (5)
O3—Gd1—O1—C5	10.5 (3)	C2—N1—C3—O4	−176.8 (3)
N3^i^—Gd1—O1—C5	170.5 (3)	C2—N1—C3—N2	3.9 (5)
N3—Gd1—O1—C5	79.2 (3)	C3—N2—C4—C1	−2.7 (5)
N4—Gd1—O1—C5	138.3 (3)	C2—C1—C4—N2	0.2 (5)
N4^i^—Gd1—O1—C5	−2.7 (4)	C5—C1—C4—N2	−179.2 (3)
O1—Gd1—O3—C2	−23.0 (3)	Gd1—O1—C5—O2	−176.9 (2)
O1^i^—Gd1—O3—C2	67.3 (3)	Gd1—O1—C5—C1	3.6 (5)
O3^i^—Gd1—O3—C2	23.1 (3)	C4—C1—C5—O2	−15.4 (5)
N3^i^—Gd1—O3—C2	171.5 (2)	C2—C1—C5—O2	165.2 (3)
N3—Gd1—O3—C2	−134.3 (3)	C4—C1—C5—O1	164.1 (3)
N4—Gd1—O3—C2	−90.5 (3)	C2—C1—C5—O1	−15.2 (5)
N4^i^—Gd1—O3—C2	149.8 (3)	C17—N3—C6—C7	2.5 (5)
O1—Gd1—N3—C6	−104.0 (2)	Gd1—N3—C6—C7	−163.2 (3)
O1^i^—Gd1—N3—C6	8.9 (3)	N3—C6—C7—C8	0.3 (6)
O3^i^—Gd1—N3—C6	161.3 (2)	C6—C7—C8—C9	−2.0 (6)
O3—Gd1—N3—C6	−36.1 (2)	C7—C8—C9—C17	0.9 (5)
N3^i^—Gd1—N3—C6	108.0 (3)	C7—C8—C9—C10	−178.9 (4)
N4—Gd1—N3—C6	−175.2 (3)	C8—C9—C10—C11	177.3 (4)
N4^i^—Gd1—N3—C6	42.3 (2)	C17—C9—C10—C11	−2.5 (6)
O1—Gd1—N3—C17	90.3 (2)	C9—C10—C11—C12	−0.4 (7)
O1^i^—Gd1—N3—C17	−156.8 (2)	C10—C11—C12—C13	−177.2 (4)
O3^i^—Gd1—N3—C17	−4.4 (3)	C10—C11—C12—C16	2.5 (6)
O3—Gd1—N3—C17	158.2 (2)	C16—C12—C13—C14	−0.5 (6)
N3^i^—Gd1—N3—C17	−57.7 (2)	C11—C12—C13—C14	179.1 (4)
N4—Gd1—N3—C17	19.1 (2)	C12—C13—C14—C15	1.6 (6)
N4^i^—Gd1—N3—C17	−123.4 (2)	C16—N4—C15—C14	−2.3 (5)
O1—Gd1—N4—C15	64.5 (2)	Gd1—N4—C15—C14	162.0 (3)
O1^i^—Gd1—N4—C15	−7.1 (3)	C13—C14—C15—N4	−0.2 (6)
O3^i^—Gd1—N4—C15	−20.2 (2)	C15—N4—C16—C12	3.4 (5)
O3—Gd1—N4—C15	128.4 (2)	Gd1—N4—C16—C12	−161.8 (3)
N3^i^—Gd1—N4—C15	−98.7 (3)	C15—N4—C16—C17	−176.8 (3)
N3—Gd1—N4—C15	176.8 (3)	Gd1—N4—C16—C17	18.0 (3)
N4^i^—Gd1—N4—C15	−139.2 (3)	C13—C12—C16—N4	−2.1 (5)
O1—Gd1—N4—C16	−131.1 (2)	C11—C12—C16—N4	178.2 (3)
O1^i^—Gd1—N4—C16	157.20 (19)	C13—C12—C16—C17	178.2 (3)
O3^i^—Gd1—N4—C16	144.2 (2)	C11—C12—C16—C17	−1.5 (5)
O3—Gd1—N4—C16	−67.3 (2)	C6—N3—C17—C9	−3.7 (5)
N3^i^—Gd1—N4—C16	65.6 (2)	Gd1—N3—C17—C9	162.9 (2)
N3—Gd1—N4—C16	−18.8 (2)	C6—N3—C17—C16	174.8 (3)
N4^i^—Gd1—N4—C16	25.13 (19)	Gd1—N3—C17—C16	−18.6 (4)
Gd1—O3—C2—N1	−158.7 (2)	C8—C9—C17—N3	2.0 (5)
Gd1—O3—C2—C1	22.2 (5)	C10—C9—C17—N3	−178.1 (3)
C3—N1—C2—O3	174.5 (3)	C8—C9—C17—C16	−176.4 (3)
C3—N1—C2—C1	−6.2 (5)	C10—C9—C17—C16	3.4 (5)
C4—C1—C2—O3	−176.9 (3)	N4—C16—C17—N3	0.3 (4)
C5—C1—C2—O3	2.5 (5)	C12—C16—C17—N3	−179.9 (3)
C4—C1—C2—N1	3.9 (4)	N4—C16—C17—C9	178.8 (3)
C5—C1—C2—N1	−176.7 (3)	C12—C16—C17—C9	−1.4 (5)

Symmetry codes: (i) −*x*+1, *y*, −*z*+1/2.

### Hydrogen-bond geometry (Å, °)

**Table:**

*D*—H···*A*	*D*—H	H···*A*	*D*···*A*	*D*—H···*A*
N1—H1···O2^ii^	0.86	2.04	2.898 (3)	178
N1—H1···O1^ii^	0.86	2.60	3.160 (4)	124
N2—H2···N2^iii^	0.86	1.81	2.669 (5)	174
O5—H5A···O4^iv^	0.85	2.14	2.970 (4)	164
O5—H5B···O2^v^	0.85	2.14	2.985 (4)	173

Symmetry codes: (ii) *x*, −*y*, *z*−1/2; (iii) −*x*+1/2, −*y*−1/2, −*z*; (iv) −*x*+1/2, −*y*+1/2, −*z*; (v) *x*, *y*+1, *z*.
